# Epicardial Implantation of a Micra™ Pacemaker in a Premature Neonate with Congenital Complete Heart Block

**DOI:** 10.19102/icrm.2024.15012

**Published:** 2024-01-15

**Authors:** Farida Karim, Daniel Peck, Shanti Narasimhan, Nicholas H. Von Bergen

**Affiliations:** 1 Pediatric Cardiology Department, Masonic Children’s Hospital, University of Minnesota, Minneapolis, MN, USA; 2 Pediatric Cardiology Department, The University of Wisconsin School of Medicine and Public Health, Madison, WI, USA

**Keywords:** Congenital complete heart block, Micra™, pacemaker therapy, transvenous pacemaker

## Abstract

Pacemaker implantation in neonates can be challenging due to their small size. Even pulse generators adapted for pediatric patients, such as the Microny device (Abbott, Chicago, IL, USA), are proportionately large in comparison to the size of the smallest newborns. Due to anatomic considerations, such as small vascular and ventricular sizes, leadless pacemakers and transvenous implantation in the youngest neonatal population remain unsuitable. Even so, the desire for leadless devices has prompted the industry to create the smallest pacemakers available. Adapting the smaller Micra™ transcatheter leadless pacing system (Medtronic, Minneapolis, MN, USA) for an epicardial pacing application may be advantageous to the smallest patients. This case illustrates the use of a Micra™ device modified with a header block to serve as the pulse generator in a ventricular epicardial pacing system for a 1-day-old, 2.68-kg patient with complete heart block.

## Introduction

Congenital complete heart block (CCHB) can be challenging to manage both prenatally and postnatally. CCHB has a reported overall incidence of 1:20,000 live births and carries a high risk of morbidity.^1–4^ Current guidelines outline pacemaker implantation as a class I recommendation for patients with CCHB for indications including, but not limited to, symptomatic bradycardia and a rate of <60–70 bpm in the setting of complex congenital heart disease or a rate of <50 bpm with a structurally normal heart.^5,6^

Neonatal pacemaker implantation is technically difficult, particularly in low-birth-weight premature newborns. Due to unique considerations, including the small physical size of these patients and the proportionately large size of pulse generators, the physiologic changes associated with birth, and the small vessel size, transvenous implantation is difficult or impossible.^7,8^ In these patients, epicardial lead placement is the standard approach. Even so, pulse generators adapted for pediatric patients, such as the Microny device (Abbott, Chicago, IL, USA), are proportionately large in comparison to the size of the smallest newborns.

Longer-term concerns include the effect that infant growth has on the leads and the associated lead–myocardial interface, which can result in a high incidence of eventual lead malfunction.^7,8^ The challenges, risks, and complications associated with pacemaker leads led to leadless pacemaker development.^9^ While the use of this relatively new technology has been well described in adult patients, transvenous implantation in pediatric patients is more limited. Often, it may not be appropriate due to the size of the delivery systems in comparison to the heart and vessels and the potential challenges with removal.^10,11^

The Micra™ transcatheter leadless pacing (TLP) system (Medtronic, Minneapolis, MN, USA) is the world’s smallest commercially available pacemaker, weighing 1.75 g with a volume of 0.8 mL. Though designed for transvenous implantation in adults, the size of the Micra™ pulse generator makes it uniquely suited to the smallest pediatric patients. Medtronic has modified the Micra™ pacemaker generator to provide a header block for epicardial pacing using a standard bipolar epicardial lead. The authors contacted Medtronic for use of this adapted Micra™ device in the newborn described below. This pacemaker was approved under “Emergency Use” authorization as the device has yet to undergo review by the U.S. Food and Drug Administration (FDA).

## Case presentation

A small-for-gestational age male fetus was prenatally diagnosed with CCHB at 22 weeks of gestation in the setting of maternal anti-Sjögren’s syndrome A/B (SSA/SSB) antibodies. The fetus developed a pericardial effusion and cardiomegaly with a heart rate <50 bpm. The delivery was expedited due to his worsening effusion. Given his low heart rate and small patient size, plans were made to employ an epicardial pacemaker using the Micra™ transvenous pacemaker system adapted to function with a standard bipolar epicardial lead. As this adapted device has not been reviewed by the U.S. FDA, we received parental consent and institutional review board approval for its use in this case.

The newborn was delivered via cesarean section at 35 weeks and 2 days of gestation, weighing 2.68 kg. Complete heart block was confirmed with a ventricular rate of 30–40 bpm and atrial rates as high as 180–195 bpm. Echocardiography also demonstrated biatrial and biventricular dilation with qualitatively normal systolic function.

After delivery, the baby developed worsening lactic acidosis due to poor cardiac output and required endotracheal intubation and sedation. Temporary transcutaneous pacing at 70 bpm was initiated in addition to isoproterenol and epinephrine infusions. A trial of transcutaneous pacing was attempted, but the intrinsic heart rate did not improve significantly and remained at 40–50 bpm. The decision was made to proceed with surgical permanent pacemaker implantation at 10 h of life.

**Figure 1:**
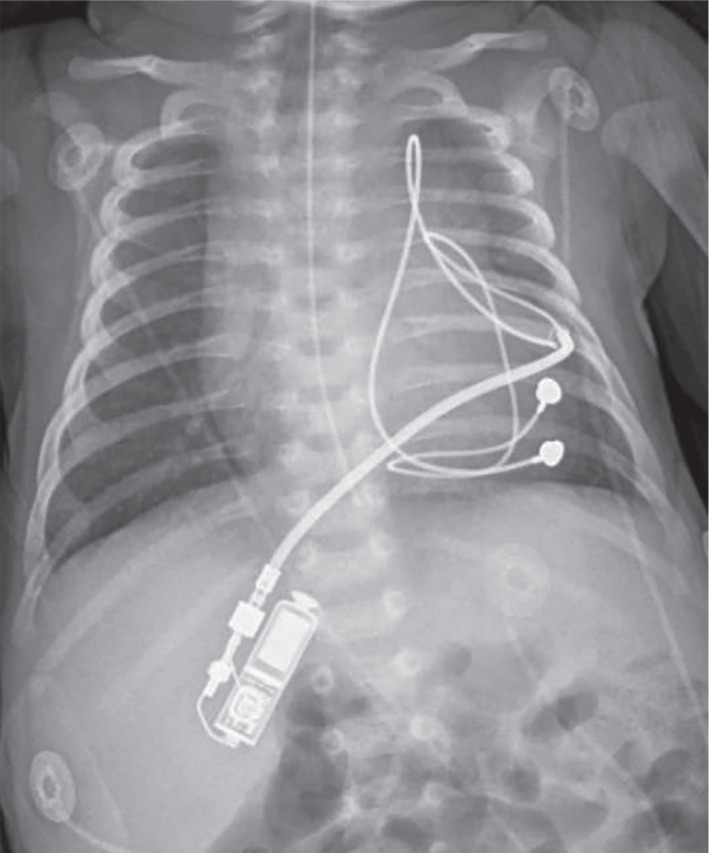
Chest radiography showing subxiphoid placement of the Micra™ implantable pulse generator connected to epicardial leads.

The patient was placed under general anesthesia and prepped in a sterile fashion. A lower median sternotomy was done. A CapSure 4968 (Medtronic) epicardial bipolar lead was placed over the lateral wall and apex of the left ventricle. This lead was found to have appropriate pacing and sensing without phrenic nerve stimulation. The pacing threshold at the time of implantation was 2 V at a pulse duration of 0.4 ms. The lead was then connected to the Micra™ implantable pulse generator (IPG), which was later placed in a subrectus pocket in the right subxiphoid area **(Figure 1)**. The initial pacemaker setting was VVI at 100 bpm, though the rate was adjusted throughout the hospital stay based on presumed heart rate needs.

In the immediate postoperative period, the infant developed sudden rapid inspirations while on neurally adjusted ventilatory assist. This was initially interpreted as the result of phrenic nerve stimulation by the IPG. However, with further evaluation, it was discovered that ventricular pacing inappropriately activated the neurally adjusted ventilatory assist sensor, prompting a ventilator-assisted breath, and the rapid inspirations ceased with the adjustment of the respiratory support.

Postoperatively, the baby developed a minor superficial wound infection and was treated successfully with antibiotics. There were no major complications related to the epicardial implantation of the pacing lead and Micra™ device. The threshold for the pacemaker at the time of discharge was 2.25 V @ 0.4 ms. The estimated longevity of the pacemaker based on discharge settings was 3.9 years. Given that the Micra™ device provides only bipolar sensing and pacing, unipolar pacing settings were not evaluated. The total hospital stay was 32 days, including 19 days in the cardiac intensive care unit, mostly due to slow feeding and weight gain. A discharge echocardiogram showed a mildly dilated right ventricle with normal systolic function and normal left ventricular size with low-normal systolic function. Afterload reduction with captopril was initiated. The patient was seen in the outpatient cardiology clinic 3 weeks after discharge and was doing well clinically with unchanged echocardiographic findings.

## Discussion

This is the first case description of the use of a Micra™ TLP system adapted to an epicardial pacing system for the management of neonatal CCHB secondary to maternal lupus. This modified device was requested from Medtronic for this premature newborn due to his small size and presumptive need for permanent epicardial pacing.

Historically, the Microny device has been the smallest available pacemaker, with a volume of 5.9 mL. It was therefore often selected for neonates who required permanent pacing. Even so, epicardial pacing with this and other small devices can be challenging in neonates, and complications were more frequent in patients who either weighed <4 kg and/or were <5 days old.^12–14^

The Medtronic Micra™ model MC1VR01 MR is a leadless single-chamber implantable transcatheter pacing system that uses CareLink (Medtronic) remote monitoring and can provide rate-responsive pacing of the right ventricle with a battery life of up to 12 years at standard (adult) settings. This device was designed for transcatheter implantation in the right ventricle using a 23-French introducer sheath and, as such, is substantially smaller (volume, 0.8 mL) than prior pulse generators.

**Figure 2:**
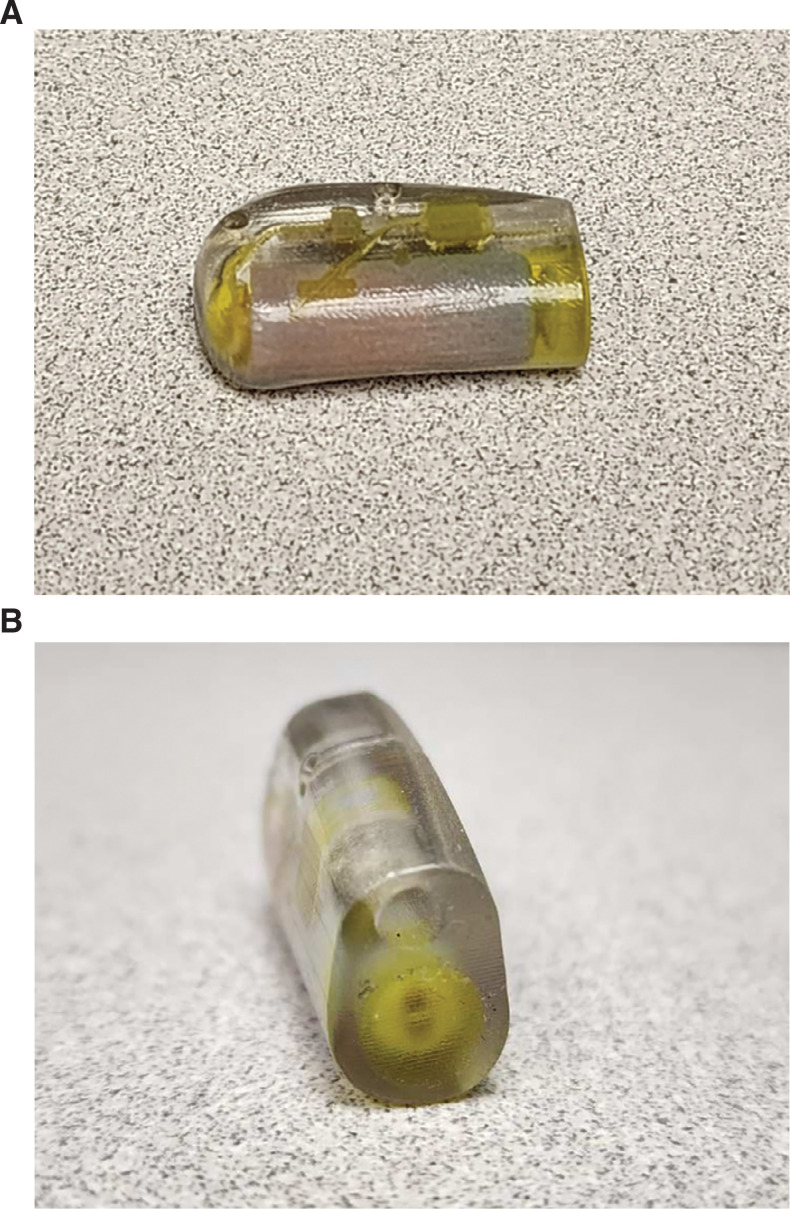
**A and B:** Two views of a Medtronic implantable pulse generator similar to the one implanted in our patient.

**Figure 3:**
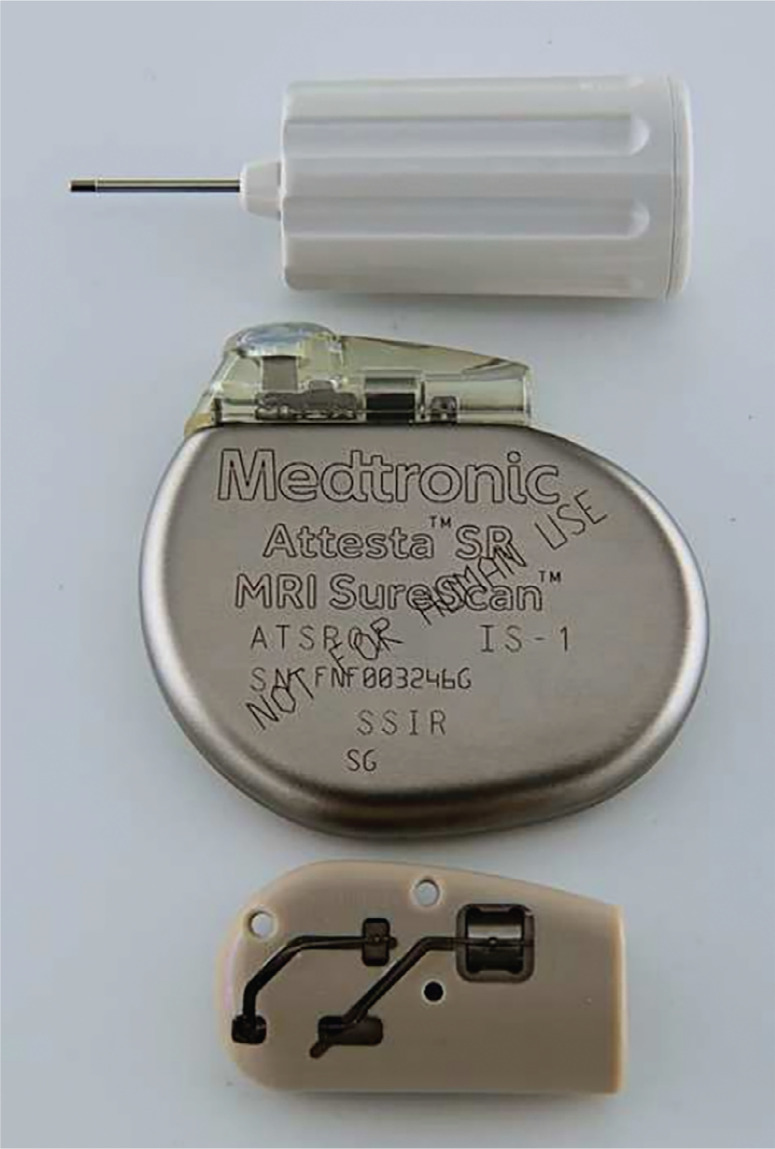
An image of a Medtronic implantable pulse generator pictured with a standard pacemaker and the pacemaker torque wrench for size comparison.

The adaptation of a header block to allow the Micra™ device to function as an epicardial pacing system **(Figures 2 and 3)** using a standard bipolar epicardial lead provided the advantages of a small device with the functionality and potential longevity of the Micra™ device. In this newborn weighing 2.68 kg, the small pacemaker size provided a substantial advantage over other devices. At the same time, the advantage of a small pacemaker size may be somewhat mitigated by the potential disadvantage of having a bipolar system that is unable to adjust to unipolar pacing or sensing should bipolar functions fail. We also appreciate the lack of data regarding the effectiveness and/or the optimal settings for rate-responsive pacing in a neonate with this device.

## Conclusion

Implantation of a Medtronic Micra™ pacemaker modified with a header block to allow epicardial pacing with a standard epicardial lead was effective in managing CCHB in a 2.68-kg neonate. This small pacemaker adapted with an epicardial lead provided a substantial advantage, given the size-related limitations to permanent pacing in this neonate. Further studies to determine safety and efficacy are warranted.
